# Experiences and perceptions about undergoing mammographic screening: a qualitative study involving women from a county in Sweden

**DOI:** 10.1080/17482631.2018.1521256

**Published:** 2018-09-14

**Authors:** Maria Norfjord Van Zyl, Sharareh Akhavan, Per Tillgren, Margareta Asp

**Affiliations:** a Division of Public Health Sciences, School of Health, Care and Social Welfare, Mälardalen University, Västerås, Sweden; b Evaluation and Analysis Unit, The National Board of Health and Welfare, Stockholm, Sweden; c Division of Caring Sciences, School of Health, Care and Social Welfare,Mälardalen University, Västerås, Sweden

**Keywords:** Experiences, focus groups, mammographic screening, participation, perceptions, public health, qualitative study

## Abstract

**Purpose:** An organized population-based mammographic screening programme aims for an early detection of potential breast abnormalities so that treatment can commence. Continuous participation and a high attendance rate are vital for an effective programme. It is important to understand the underlying reasons for participation in mammographic screening, should there be factors that are amendable within reason and could be adjusted. Therefore, the invited women are valuable sources of information. This study aimed at describing the experiences and perceptions about mammographic screening of women from three municipalities in a Swedish county.

**Method:** Six semi-structured focus-group discussions, each with four to five participants, were held. Content analysis was then conducted.

**Results: **The screening procedure, such as staff professionalism, was covered. Other people’s opinions and the woman’s own understanding affected the women’s decisions on whether or not to undergo the procedure. Structural conditions, such as travel time and financial issues, were sources of concern. However, the offer to perform mammographic screening was perceived with gratitude.

**Conclusions:** Structural conditions, risk and time perceptions, the screening procedure, attitudes towards undergoing it and appreciation of its benefit may influence the women’s continuous willingness to be screened, which in turn may affect public and individual health.

## Introduction

Breast cancer is a major public health concern as the most common type of cancer among women worldwide. In 2012, it accounted for a quarter of the cancers in women, with an estimated 14.7% of the global deaths from all types of cancer, excluded non-melanoma skin cancer (Ferlay et al., 2015). This translates to approximately 500 000 women dying of breast cancer globally in 2012 (Ferlay et al., 2015; Stewart & Wild, 2014). In Sweden, 7558 women were diagnosed with breast cancer in 2016, accounting for approximately 30% of cancers among Swedish women (Swedish Cancer Society, 2018). Early diagnosis is important since it facilitates early treatment and may save lives (World Health Organization, 2017). Mammographic screening, which is an x-ray examination, is one option for an early detection of potential breast malignancies and consequently, for the commencement of treatment (Vainio & Bianchini, 2002; Wilson & Jungner, 1968). The pros and cons of screening for breast cancer have been debated; negative effects, such as the risk of unnecessary treatment due to over-diagnosis (Jørgensen, Keen, & Gøtzsche, 2011; Løberg, Lousdal, Bretthauer, & Kalager, 2015), have been mentioned, while a mortality reduction has been a counter argument (Duffy et al., 2002; Independent UK Panel on Breast Cancer Screening, 2012). However, the organized population-based mammographic screening programme is perceived as effective, and the majority of the countries in the European Union (except three) implemented it in 2016 (Ponti et al., 2017). For an organized population-based screening programme to be offered, it should be (among other things) cost-effective, which requires a high-attendance rate (National Board of Health and Welfare, 2014a; Törnberg, Lidbrink, & Henriksson, 2014). In Sweden, mammographic screening is offered to all female residents between the ages of 40 and 74 (National Board of Health and Welfare, 2014b); the approximately 80% national attendance rate (Deandrea et al., 2016; National Board of Health and Welfare, 2014b) is aligned with the national recommendation for an attendance rate of at least 80% (Official Reports of the Swedish Government, 2009). Nevertheless, the attendance rates in the counties vary from 71% to 90% (Swedish Breast Cancer Association, 2015); differences have also been found among areas in cities, with attendance rates ranging from 37% to 82% (Zackrisson, Lindström, Moghaddassi, Andersson, & Janzon, 2007). An integrative literature review of earlier studies regarding factors influencing mammographic screening participation has identified a range of reasons, from individual circumstances to more general conditions, such as socioeconomic status and age (Edgar, Glackin, Hughes, Mary, & Rogers, 2013), with many of the studies focusing on non-participation (Achat, Close, & Taylor, 2005; Borda et al., 2011; Brustrom & Hunter, 2001; Fallowfield, Rodway, & Baum, 1990; Gierisch et al., 2009; Johansson & Berterö, 2003; Lagerlund, Hedin, Sparén, Thurfjell, & Lambe, 2000; Lagerlund, Widmark, Lambe, & Tishelman, 2001; Pietrzak, Godlewski, & Adamczak, 2011; Watson-Johnson et al., 2011). This study focuses on the attendees as it could be assumed that continuous participation in mammographic screening is important. The invited women are thus valuable sources of information. They base their decisions to undergo the screening on objective and subjective reasons, which may be important to understand in order to maintain an acceptable participation rate. To the best of the authors’ knowledge, only one previous study in Sweden has conducted similar research (Willis, 2008). The findings of a survey conducted in a Swedish county with only one fixed mammographic facility indicated that age and distance may be associated with non-participation in mammographic screening (Zidar, Larm, Tillgren, & Akhavan, 2015). How can the meaning of these and other conditions for women who undergo the screening be understood in a deeper way? This study aimed to describe the experiences and perceptions about mammographic screening of participating women from three municipalities in a Swedish county.

## Material and method

A descriptive qualitative interview study was conducted, with six focus groups of women. A qualitative content analysis was performed on the data.

### Setting

Three out of the 10 municipalities in a county in central Sweden were chosen, as all shared the same single mammographic screening facility. The selection of the municipalities was based on a previous study’s results (Zidar et al., 2015). These municipalities (A, B and C) displayed the lowest (A) and the highest (C) percentages of non-participation. The third municipality (B) was chosen since it showed an exception to an observed trend (although not statistically tested) of lower participation rate among municipalities that were located farthest from the municipality with the screening facility (see Table I), which was also the reference city (Zidar et al., 2015).

**Table 1.** Sampling result.

	Municipality A		Municipality B		Municipality C	
Attendance rate	84.4 per cent		80.4 per cent		75.6 per cent	
Distance from reference city	20–40 km		60–80 km		60–80 km	
Focus group	A 1	A2	B1	B2	C1	C2
Number of participants	4	4	5	4	5	5
	Same workplace		Age group 70 +	Same workplace	Same residential area	Same workplace
	Same type of job		Different types of jobs,when working	Similar types of jobs	Different types of jobs	Similar types of jobs
	Acquainted		Not acquainted	Some acquainted	Well acquainted	Acquainted

Note. Attendance rate and distance from reference city (Zidar et al., 2015)

#### Selection of participants

Snowball sampling (Erickson, 1979) was conducted due to difficulties in recruiting participants. One contact person was initially asked if she knew any resident in each of the selected municipalities, who could be interested in functioning as a gate keeper. This resulted in finding one gate keeper in one of the municipalities; the other gate keepers for the two remaining municipalities were found by the first author contacting (via telephone and mail) different interest associations located in these municipalities. All the gate keepers were mailed, by the first author, the study’s aim and the contact details for further questions. Each gate keeper contacted people at her workplace or in the interest association, asking if anyone was interested in participating in a focus-group discussion about mammographic screening. Interested persons gave their e-mail addresses, which were forwarded to the main author, so a direct contact could be established to distribute an information letter, a consent form and a short questionnaire to obtain a simple socio-demographic profile of each participant. Through this direct contact, each interested person was asked if she in turn knew anyone who might be interested in participating in a focus-group discussion as well. Participants were eligible if they resided in one of the three chosen municipalities, were 40 to 74 years old, were fluent in Swedish and had been invited to a mammographic screening in the county’s facility. Initially, 28 participants were included, but one non-attendee was later excluded. Hence, the sampling resulted in six focus groups consisting of 27 participants in total, with two groups per municipality (see Table 1).

The signed consent form and the questionnaire regarding the socio-demographic profile had to be brought to the focus-group discussion. The socio-demographic profiles (see Table 2) is only used in this study to give brief descriptions of the participants’ backgrounds.

**Table 2.** Socio-demographic characteristics of the participants.

Variable	*N*
Age range (median age: 60 years, range: 42–74 years)	
40–49	5
50–59	8
60–69	9
70–74	5
*Total*	*27*
Marital status	
Single	4
Cohabiting/married	18
Divorced/widowed	5
*Total*	*27*
Number of children	
0–1	3
2	14
3+	10
*Total*	*27*
Level of education	
Primary	2
Secondary	9
Tertiary	16
*Total*	*27*
Country/region/continent of origin	
Sweden	23
Scandinavia	1
Europe	2
Not Europe	1
*Total*	*27*
Any of the parents born in another country	
No	18
Yes	8
No answer	1
*Total*	*27*
Participation	
Participant of mammographic screening	25
Irregular participant of mammographic screening	2
*Total*	*27*

#### Data collection

The preparation and the interviews followed the focus-group methodology (Krueger & Casey, 2015) to create favourable and structured conditions concerning the interview guide, the number of focus groups and the group size. Each group consisted of four to five participants. This small group type has gained popularity as it can be perceived as more comfortable for participants (Krueger & Casey, 2015; Peek & Fothergill, 2009), while extracting the most out of the topics discussed (Peek & Fothergill, 2009). A semi-structured interview guide that followed a question route (Krueger & Casey, 2015) was used. The following are some examples of the questions: “What do you think of when you hear the term mammographic screening?” “Any thoughts about [the fact] that the invitation is sent out every second year?” “Which is the most important factor for undergoing mammographic screening?”

Before each focus-group discussion, the participants’ rights were repeated orally, and then the signed consent forms were gathered. The first author moderated the discussions; a colleague assisted by taking notes and summarizing the discussions. This procedure allowed the participants to correct, elaborate on or clarify the statements that had been expressed during the discussions. Each discussion lasted 50–70 min and was audio recorded and transcribed verbatim. Ethical approval was obtained from the Uppsala University Regional Ethical Review Board (Dnr. 2015/393) to comply with the Swedish Ethical Review Act and consequently, the Declaration of Helsinki (World Medical Association, 2001).

#### Data analysis

A qualitative content analysis with an inductive approach (Graneheim, Lindgren, & Lundman, 2017; Graneheim & Lundman, 2004) was conducted by identifying meaning units, followed by condensing, coding and abstraction into subcategories to classify the results later into categories (see Table 3). Each focus group discussion was read through several times and analysed separately at first. Meaning units were marked by using the “New Comment” review function in Microsoft Word, with a short explanatory text to facilitate remembrance of events or thoughts by and for the first author, depending on the content. Different fonts were allocated for each group to facilitate recognition of the municipality, as well as the group. After separate analyses of the groups, the group discussions for each municipality were merged, with different colours assigned to each municipality to facilitate reviews of the original transcripts, if needed, during the analysis process. Eventually, all the municipalities’ focus group discussions were merged and treated as a unit due to similar accounts in the material, regardless of the municipality of residence. The last author read through and discussed the first author’s subcategories and categorization, and consensus was achieved to strengthen credibility.

**Table 3.** Example of the analysis.

Meaning unit	Condensed meaning unit	Code	Category
“… since I have children, it always becomes a puzzling [putting the pieces of the puzzle] together, and then one shall go there and then one must get rid of the car and be on time (laughs) and in the city [where the mammographic facility is located], it looks as it does (laughs) when one shall go there. So, it is not just to go there and back again as … so it is [takes] a bit of planning before … [going]”. (Participant 1, FG A)	It always entails putting the pieces of the puzzle together when one has children, being on time and planning where to get rid of the car. It is not just going there and back.	Practical issues and alternative solutions	*Required and recurring planning*
“Yes, it is more difficult to go to the city, like parking and pay[ing] for the parking and all those things. And then there is a distance to travel there and that feels … most of the time I am so economical and environmentally aware, so I want to do several things in the city then”. (Participant 3, FG A)	Want to do several things at the same time, being economical and environmentally aware, and it is a distance to travel to the city, park and pay for the parking.	Inconvenient circumstances	

## Findings

The findings from the focus groups were classified into four categories (see Table 4). To illustrate the categories, some quotes from the participants are provided. An ellipsis (…) indicates omitted words or sentences, a double slash (//) stands for hesitation or a pause, and text enclosed in square brackets shows an author’s comment.

### Insecurity surrounding the screening procedure

The category called *insecurity surrounding the screening procedure* contains three subcategories that address the events before, during and after the examination itself.

#### Thoughts and feelings concerning examination

This includes the physical contact with the machine and the staff. Getting undressed in the small changing booth and waiting for unknown healthcare staff to handle the participant’s breasts are perceived as unpleasant necessities. Having a woman’s breasts handled is occasionally painful as it includes pressing and squeezing them onto a cold X-ray machine.

Participant 9 (P9): … there is nothing you can do about feeling exposed because it is that kind of … examination you must undress for (Focus group A).

**Table 4.** Categories and subcategories.

Category	Subcategory
Insecurity surrounding the screening procedure	Thoughts and feelings concerning examination
	Uncertainty when waiting
	Lack of information
Participation as a norm	Impact of other people’s stories and opinions
	Understanding and prioritization
Required and recurring planning	Practical issues and alternative solutions
	Importance of finances
Gratitude and respect for mammographic screening	An appreciated benefit
	Awareness of the value of mammographic screening

The importance of meeting professional staff and the reception are expressed, as the circumstances around and during the examination itself is perceived as being abnormal and can evoke nervousness. This reaction is not always due to the examination itself, but can be related to the results of the examination. Some of the participants have been called back for a second examination due to difficulties in interpreting the x-ray images. This case might be because of a technical issue; nevertheless, it causes stress and fear of potential malignancies.

P19: … she [the staff member] said it, and she was very cautious to point out that ‘if it is written in the letter that you have to come back because the images have to be retaken, it is not dangerous because images are sometimes of poor quality’ (Focus group C).

#### Uncertainty when waiting

The mail with the screening result is a source of tension; the shorter the time after the examination when the results are received, the better it is. Regardless of experiences of being called back or not, most participants feel uncertain about what the results may entail, which causes worry.

P24: … sometimes you receive notification the day before you are about to go away or do something. And then I think, ‘But God, do I dare open this. Then perhaps … my trip might be ruined’ (Focus group C).

#### Lack of information

Sometimes, not knowing and informational inconsistencies also cause confusion about the expected date for the results. Contradictory information about being allowed to take the initiative to contact the staff regarding mammographic screening after the upper age limit for the invitation has passed also causes frustration.

P10: Last time I underwent mammo//mammography, that is, for (Someone coughs) two years ago, then one had the possibility to//then they said//then I know that we asked, ‘When we get too old, can we participate?’ (P11: yes?) and ‘Yes, it is just to phone us and book an appointment’. Then we asked the same thing again this year … and then it is required, then one cannot, then one needs a referral from the doctor … (Focus group B).

Trust in screening concerns a range of issues, such as whether the biannual invitation interval is seldom or frequent. Additionally, the validity of the age range is questioned, which can express the lack of information.

### Participation as a norm

For most participants, the decision to undergo screening is an obvious one. An unquestionable acceptance is perceived as a natural part of life. The *participation as a norm* category reflects how the participants view their own and others’ decisions to undergo screening.

#### The impact of other people’s stories and opinions

Where participation is encouraged and expected, it might result in accepting the invitation. Some of the participants express participation as an automated response.

P10: I have never considered not to participate!

Unidentified voice: No!

P13: It’s a given (Focus group B).

If asked for guidance by someone who is hesitant to accept the invitation to the mammographic screening, a participant would advise acceptance.

P2: … I would promote it, but yes, I would start the conversation with, ‘Well, what are you thinking now//what are you going to do?’ (Focus group A).

However, the participants mention respect for the individual’s right to refrain from mammographic screening. Additionally, cultural influences may have an impact on the decision. For example, one participant expressed the belief that breast cancer cannot be prevented; hence, a person must wait until it is a fact. Another cultural effect can be the family tradition to have the examination.

P6: But then, I also think you get influenced by your mother//you think like that (P9: yeah)//that you just undergo [the screening] (Focus group A).

#### Understanding and prioritization

The perception of the importance of undergoing the screening sometimes collides with something else that takes priority. One way to mitigate this conflict is to conduct self-examination of the breasts.

P17: There is time when it is needed//health is the most important, not time [that it takes to have it done] (Focus group B).

According to all participants, the main and most important reason for undergoing mammographic screening is to detect any malignancies in order to start treatment early. The subjective risk perception of breast cancer, such as hereditary risk, as well as the idea of the correlation between breast size and susceptibility to breast cancer, is another reason to have the examination.

### Required and recurring planning

The *required and recurring planning* category addresses conditions beyond the individual’s direct control and depends on the set-up of society, as well as healthcare. Participation in mammographic screening involves planning before the examination and is connected to time, transportation, costs, the work situation and child care.

#### Practical issues and alternative solutions

The participants pay attention to this topic because of its impact on their daily lives. For the majority, it includes special planning. Work must be considered by applying for a day’s leave or using a vacation day to undergo the screening. Going through these measures makes it important to try to combine other errands with the visit to the mammographic facility. Planning also includes the means of and arrangements for transportation, time and weather conditions, child care and parking or the transits needed upon arrival in the city. The distance to the facility engages all participants because it poses a hurdle, and a recurring topic is the mobile mammographic screening unit that was decommissioned in 2006.

P10: … before, we had the mobile unit that came here (Unidentified voice: mm; P11: yes) was absolutely fantastic mammographic mobile unit//stationed below the hospital//then we didn’t need to travel. (Focus group B).

Most participants miss the mobile unit due to the convenience of not having to plan, and they request it back. However, some participants have negative experiences, mainly due to problems with inconsistent x-ray images, resulting in being called back. This situation involves both a feeling of concern and having to visit the main mammographic facility. Thus, some participants prefer to travel a bit longer for better x-ray images and minimize the risk of being called back. Some participants suggest coordinating a carpool with colleagues when it is time for mammographic screening. Others think that the responsibility to facilitate carpooling ought to be initiated by the mammographic facility or the county council. Nonetheless, alternative solutions are appreciated, such as having the possibility to change the schedule and the offer of evening appointments.

##### Importance of finances

Other consequences of residing farther away from the central mammographic facility are the added costs in the form of petrol or public transportation fees and if employed, a loss of some income.

P14: … it takes a whole day, now I can decide on my [schedule for the] day, but those who work, and then I can think it takes a very long time (Unidentified voice: mm) and that it is a bit unequal//because those who live in the city, they have it close … don’t have to pay for the trip … (Focus group B).

### Gratitude and respect for mammographic screening

The *gratitude and respect for mammographic screening* category reflects perceptions about the offering of the organized population-based mammographic screening programme, both from a public point of view and the impact on the individual.

#### An appreciated benefit

The invitation to the screening should be utilized. Additionally, the service should be based on invitation, so the participants do not have to think about it. Some participants think that this offer shows the welfare state taking care of its residents. In addition to the invitation letter, it could be valuable to receive further information from other sources about the benefits of undergoing the screening. Talking about participating in the screening with colleagues and friends is one way to promote it, and by using different forums, the information can reach more people. By having more discussions, the importance of participation is acknowledged, and its benefit is not taken for granted.

P8: Yes, but this is a benefit we have//that has to be utilized … Absolutely (Focus group A).

#### Awareness of the value of mammographic screening

The recognition of the screening as a life saver is expressed as having a meaningful effect on individuals, as well as relatives.

P22: Yes, I think it is a security since we have it [breast cancer] in the family, so I think it is a security (Several others: mmm) … (Focus group C).

The value is also discussed from the age perspective. Some participants express their frustration at no longer being invited after the age of 74.

## Discussion

This study focuses on participating women. The results identify structural conditions, risk and time estimation, the screening procedure, the appreciation for the benefit, attitudes towards participation and having an influence when deciding to undergo mammographic screening. The other study conducted in Sweden (Willis, 2008), with participants as the sources of information, reports the same reasons, such as the importance of being invited and not having to call for an appointment. Another similarity is the automated response to participate when invited, but differing to some extent in the expression of the underlying reason for accepting the invitation. Willis (2008) cites the reason as more a reflection of the state’s role in deciding what is best, whereas the current study finds that it is more an indicator of a norm, without identifying the originator. Another similar aspect is the time factor to enable an early detection of any abnormalities. Despite many similarities between the current study and that of Willis (2008), some differences emerge. The participants in Willis’ (2008) study have four facilities in their county compared with one common facility in the county involved in the current study. The former study only includes 40–49-year-old participants, whereas in the current study, the participants’ ages range from 42 to 74.

The results also corroborate previous studies’ findings (Borda et al., 2011; Gierisch et al., 2009; Johansson & Berterö, 2003; Lagerlund et al., 2001) concerning the underlying reasons for refraining from mammographic screening, which imply that when deciding to accept or decline, the reasons and considerations seem to be of a universal nature rather than exclusive to participants and non-participants, respectively. Most of the women in this study have jobs and incomes, but they still voice opinions regarding all the costs incurred in the visit and the distance that causes inconveniences. The threshold to the transition from participating to refraining is not explored here, but understanding the similarities in reasoning is worth acknowledging to promote and facilitate the choice to avail of mammographic screening, as well as other health services. This recommendation also corresponds with the public health goal to reduce inequalities (Munthe, 2008).

The categories emerging from this study that relate to participation are similar to the findings of other studies conducted in Sweden (Lagerlund et al., 2000; Manjer, Zackrisson, & Emilsson, 2016). The outcome indicates stability over time regarding the underlying reasons for deciding to undergo or refuse mammographic screening.

Structural conditions as social determinants of health have been vividly discussed (Dahlgren & Whitehead, 2007), and these are more difficult for the individual to influence directly. For instance, infrastructure, commuting schedules and the locations of facilities may have greater consequences for those who are ambivalent about continued participation. The screening procedure also receives much attention, especially the waiting times for the examination results. Many participants perceive this period as unpleasant, which could be an expression of their uncertainty about what to expect. A disempowered feeling may discourage participation if this negativity is not alleviated by healthcare professionals. A counteraction could be increased accessibility, for instance, using a context-adjusted form of patient navigators (Freeman, 2006; Natale-Pereira, Enard, Nevarez, & Jones, 2011), should concerns arise. Similar to the findings of other Swedish studies, the screening procedure is perceived as standardized, with its focus on one body type, as well as sometimes being painful and automated (Johansson & Berterö, 2003; Lagerlund et al., 2001). However, the conduct of the staff may affect this experience (Morris, 2015; Whelehan, Evans, & Ozakinci, 2017). Hence, the importance of staff’s concern for the women’s feeling of comfort in an exposed situation cannot be underestimated (Van Goethem et al., 2003). Interestingly, the attitude towards one’s own, as well as others’ decisions to participate, is often described as a certainty and may mirror the norm of acceptance when offered a benefit. However, as society changes, the norms may follow suit, so how can these potential changes be predicted and, if important to public health, be addressed?

The findings that reflect the levels that could be targets of potential interventions can be divided into three broad domains: societal, healthcare and individual. The societal domain is mainly reflected in the required and recurring planning category, where issues such as costs, work conditions and child care are dictated at a level further from the individual’s direct control. Healthcare is also represented in this category as it sets the conditions for visiting hours and flexibility concerning individual needs. The healthcare and the individual domains are identified in the insecurity surrounding the screening procedure category since it involves more direct contacts between the individual and the healthcare system, regarding both the examination itself and the routines performed in the whole process before, during and after the screening. The societal and the individual domains meet in the participation as a norm category, where part of the decision to participate may be a result of a norm set in society, in the family, among friends and in individual beliefs and actions. The gratitude and respect for mammographic screening category reflects the individual’s perception of the state as catering to the needs of the individual and society by offering certain services, such as mammographic screening to all women between 40 and 74 years of age. However, all the domains are more or less represented in all categories as they are intertwined, indicating that the decision to undergo or decline mammographic screening is complex, and more factors are considered than just “yes” or “no”. Women who undergo mammographic screening are valuable sources of information by adding their perspective before others decide to have or not have the examination, as they face the same or similar conditions as non-participants do. Therefore, it becomes of greater importance that the participating women in this study also address the practical barriers. They have decided to participate even though they all reside in municipalities that lack a local mammographic facility, and they express frustration about extra costs and planning for the visit itself. Additionally, most of the women in this study have jobs and incomes, but they still voice their opinions regarding all the costs incurred in the visit and the distance that causes inconveniences.

If these are issues raised by participants, it could be assumed that these conditions need to be considered for the future to maintain a high percentage of participants so as to justify this health service. As a universal healthcare system relies on the tax payers’ willingness to support it (Diderichsen, 1995), this perspective becomes relevant.

The municipalities included in this study are all located at different distances from the central mammographic facility. However, the participants express similar experiences and perceptions concerning obstacles to undergoing mammographic screening, irrespective of the municipality of residence. This result indicates that the importance of early detection and treatment (Willis, 2008) may supersede the inconveniences and accompanying nuisance of planning, travel, costs, recall, pain (Morris, 2015) and nervousness experienced by the participants of this study. Other studies have reported similar findings, but the difference is that the county involved in this study only has one mammographic facility. Offering alternative solutions regarding visiting hours and the possibility to reschedule may, at least for the time being, be mediating factors that are equivalent to the presence of several mammographic facilities.

## Limitations and strengths

A possible weakness of this study was its sampling method, which resulted in a homogeneous group of participants (Polit & Beck, 2010). The intention was to recruit two groups per chosen municipality to achieve a broad representation of women from different socio-demographic strata, and different means were used to recruit a more heterogeneous group of participants. For instance, posters were displayed at the local primary healthcare facilities to seek participants for the group discussions. Additionally, relevant actors from a local network were contacted for advice on whom to approach to get in contact with potential participants, but without success. The sampling resulted in homogeneity in the participants’ socio-demographic profiles, such as similarities in educational levels and ethnicity. Most of the participants had a higher level of education, and a more heterogeneous group may have resulted in other experiences and perceptions, which can only be subjects of conjecture.

In this context, the different groups varied to a certain extent regarding age, workplace, job title and municipality of residence. Other differences regarding the group constellations could also be identified among the municipalities.

Nonetheless, homogeneity alone do not define a disadvantage. It is a characteristic of a focus group as it highlights a specific common denominator (Krueger & Casey, 2015), in this case, participation in mammographic screening. For instance, some women might feel more comfortable and engage in discussions with their colleagues in the same workplace when addressing more sensitive topics such as mammography. Avoiding having participants with different levels of power positions could counteract any fear of or insecurity in expressing viewpoints (Krueger & Casey, 2015). It could also be ethically questionable to cancel the focus-group discussions because the participants who expressed their interest are not perceived as ideal enough. These participants decided to share their experiences and perceptions about a topic and reserved a time to join the discussion, which should not be taken for granted but be respected. It can be reflected upon, if and how any sharing of the same workplace or being acquainted with each other could have had an impact on the decision to attend mammographic screening, or the expressed viewpoints regarding the mammographic screening procedure. The influence from the surrounding society affecting the decision to attend mammographic screening is constantly present. For example, the State’s implicit expectation of attendance to the screening by inviting all women in a certain age group (Manjer et al., 2016). Other societal influences could include work colleagues discussing their intent to attend the mammographic screening, or friends and relatives expressing attendance as an unquestionable fact. All these actors are part of the society and existing in women’s daily lives, hence may exert implicit or explicit pressure to attend. The national attendance rate of approximately 80% in Sweden (Deandrea et al., 2016; National Board of Health and Welfare, 2014b) may partly be explained by these circumstances. Another Swedish qualitative study focusing on women attending mammographic screening (Willis, 2008) used purposive sampling (Polit & Beck, 2010) and had a more heterogenic sample. It accounted for similar findings as the present study regarding reasons to attend mammographic screening. This could indicate that some reasons to attend may be more pervading and transcend social strata.

Trustworthiness, a core concept in qualitative research, reflects the confidence in the data (Polit & Beck, 2010). By using a semi-structured interview guide and following the same question route (Krueger & Casey, 2015), dependability was strengthened even though the time span for conducting the focus-group discussions did not cover more than two months. The first author discussed the results of the analysis with the last author, and a consensus was reached, in order to strengthen the credibility of the findings (Graneheim & Lundman, 2004). Additionally, quotes from the transcribed group’s discussions are included in the paper as well as an audit trail of the analysis, to demonstrate credibility. The method used in this study has been accounted for, and the group discussions reflect commonalities among different women concerning decisions about health-related issues, such as undergoing mammographic screening. This understanding of important conditions and concerns when deciding how to respond to an invitation may be valuable in similar situations. It may be of interest in the event of being transferred to another setting. All of the above measures have been taken to increase the study’s trustworthiness and are aligned with the method described by Graneheim et. al (2017) and Graneheim and Lundman (2004).

## Ethical considerations

Focus groups may encourage participants to reveal their thoughts if the atmosphere is welcoming and non-judgemental (Krueger & Casey, 2015). When inviting the participants, they were informed that their attendance in the focus-group discussion was voluntary. It was clearly stated that the discussion would focus on participation in mammographic screening. If any woman would feel uncomfortable discussing this topic, it could be assumed that she would not sign up to join the discussion. Additionally, in the beginning of the group discussion, the moderator explained some rules of conduct, including respect for one another’s experiences and perceptions.

## Conclusions

This study represents women who have participated in mammographic screening. However, the participants’ expressed experiences and perceptions regarding mammographic screening are congruent with previous findings of studies focusing on both those who have undergone and not undergone the screening. This study also adds a geographic perspective, as the common denominator for the women is their residence in the same county, along with their shared experiences and perceptions of being invited to the only, hence pre-selected, mammographic screening facility in their area. Structural conditions, risk and time estimation, the screening procedure, the appreciation for the benefit and attitudes towards participation have impacts on the women’s decision to have the examination. These issues need to be considered, for instance, when planning for new screening services or improvements in existing ones and for infrastructural conditions, as they may influence the women’s continuous willingness to undergo mammographic screening. In turn, their willingness may affect the health of both individuals and populations.
